# Synthesis and Examination of Nanocomposites Based on Poly(2-hydroxyethyl methacrylate) for Medicinal Use

**DOI:** 10.1186/s11671-017-1881-7

**Published:** 2017-02-20

**Authors:** Olena S. Kukolevska, Igor I. Gerashchenko, Mykola V. Borysenko, Evgenii M. Pakhlov, Michal Machovsky, Tetyana I. Yushchenko

**Affiliations:** 1 Chuiko Institute of Surface Chemistry, NAS of Ukraine, 17 General Naumov Str., Kyiv, 03164 Ukraine; 2 Centre of Polymer Systems, University Institute, Tomas Bata University in Zlin, Tr. T. Bati 5678, Zlin, 76001 Czech Republic; 3 Vinnytsia National Pyrogov Memorial Medical University, 56 Pyrogov Str., Vinnytsia, 21018 Ukraine

**Keywords:** Nanocomposites, Poly(2-hydroxyethyl methacrylate), Silica, Biologically active compounds, Pore formation, Controlled release

## Abstract

Preparation of poly(2-hydroxyethyl methacrylate) (PHEMA) based nanocomposites using different approaches such as synthesis with water as the porogen, filling of polymer matrix by silica and formation of interpenetrating polymer networks with polyurethane was demonstrated. Incorporation of various biologically active compounds (BAC) such as metronidazole, decamethoxin, zinc sulphate, silver nitrate or amino acids glycine and tryptophan into nanocomposites was achieved. BAC were introduced into the polymer matrix either (1) directly, or (2) with a solution of colloidal silica, or (3) through immobilization on silica (sol-densil). Morphology of prepared materials was investigated by laser scanning microscopy and low-vacuum scanning electron microscopy. In vacuum freeze-drying, prior imaging was proposed for improving visualization of the porous structure of composites. The interaction between PHEMA matrix and silica filler was investigated by IR spectroscopy. Adsorption of 2-hydroxyethyl methacrylate and BAC from aqueous solution on the silica surface was also examined. Phase composition and thermal stability of composites were studied by the differential thermogravimetry/differential thermal analysis. Release of BAC into water medium from prepared composites were shown to depend on the synthetic method and differed significantly. Obtained PHEMA-base materials which are characterized by controlled release of BAC have a strong potential for application in manufacturing of different surgical devices like implants, catheters and drainages.

## Background

Hydrogels based on poly(2-hydroxyethyl methacrylate) (PHEMA) are interesting for application in medicine due to their chemical, biochemical and hydrolytic stability [1], high permeability for oxygen and water-soluble compounds, including metabolites, through the polymer network [2, 3], their shape stability and biocompatibility [4]. The hydroxyl and carbonyl groups on each PHEMA monomer chain determine its hydrophilic properties while the hydrophobic α-methyl groups and carbon backbone provide resistance to hydrolysis and the mechanical strength of the polymer matrix [5].

There is a high variety of established methods for the synthesis of PHEMA hydrogels, such as copolymerization [6], radiation-initiated polimerzation [7] and radical polymerization by atom transfer [8], etc. They lead to the formation of the cross-linked networks or intertwined linear homopolymers, linear copolymers or semi-interpenetrating networks (IPN) [9].

The porosity is an important feature of hydrogels for biomedical applications and depends on the synthesis conditions. Polymerization in bulk permits to obtain glassy, transparent materials which after swelling in water or other solvents remain soft and flexible. The mass transfer of low molecular weight substances in swollen gels is achieved mainly by diffusion through the spaces between macromolecular chains. Migration of large molecules is possible through reptations [10]. However, it is assumed that these gels do not have a porous structure and are “homogeneous”. When PHEMA polymerization is carried out in the presence of a solvent which is taken in the amounts below the critical concentration (up to 45 wt% for water), it results in the gels that keep their optical transparency. Moreover, the swelling propensity as well as the size of the pores will increase [11]. The main feature of the gels synthesized in the presence of the solvent is formation of the cross-links which are separated by large areas of flexible polymer chains. In terms of the soluble substance diffusion through obtained gels [11], they are generally classified as (1) “microporous” with a pore size in the range of 10–50 nm and (2) “macroporous”—with pores from 100 to 1000 nm or even bigger. Convection in these materials gradually becomes the dominant mechanism for the transport of substances. If the water content in the initial monomer mixture is higher than 45 wt%, there is a phase separation during polymerization. As a result, transparency is gradually lost with the formation of “white” gels, which are classified as heterogeneous hydrogels or sponges.

In order to improve stability and mechanical properties of PHEMA and to maintain its hydrophilicity, the IPN synthesis of this polymer with polyurethane (PU) was proposed [12]. The introduction into mixed matrix PU/PHEMA of special fillers, in particular nanosized silica with biologically active compounds (BAC) which immobilized on its surface, allows to create composites with specific pharmacological properties [13]. Adsorption modification of fumed nanosilica allows to convert BAC in highly dispersive state and to obtain their mono- and multimolecular layers on the nanoparticles surface [14]. This approach considerably slows down the migration of BAC in a polymer matrix and creates conditions for their deposition. It was found that composites based on IPN PU/PHEMA are non-toxic, do not cause any local inflammatory reactions and have antimicrobial properties [15].

The aim of this work is the synthesis of PHEMA-based materials with the controlled release of BAC. The following ways of synthesis were used: filling by nanosilica which is modified by BAC, the creation of IPN PU/PHEMA, the formation of pore with water as a porogen and combination of listed methods. In this study, the morphology of prepared materials was investigated by laser scanning microscopy (LSM) and low-vacuum scanning electron microscopy (LVSEM) methods. Interaction of PHEMA with nanosilica and adsorption of BAC on the silica surface were studied by IR and visible spectrophotometry. The features of thermooxidative destruction of composites by differential thermogravimetry (DTG)/differential thermal analysis (DTA) and the release of BAC from composites into aquatic medium were examined.

## Methods

The following materials were synthesized for structural and pharmacokinetic studies (Table 1).

**Table 1:** Composition of the studied materials

No of series	Polymer matrix	Introduced into the matrix
1	PHEMA	BAC (see Table 2)
2	PHEMA	10 or 15% of sol-densil
3	PHEMA/water	BAC
4	PHEMA/water	10 or 15% of sol-densil
5	PHEMA/water	10 or 15% of nanosilica + BAC
6	PU	10 or 15% of sol-densil
7	IPN 83%PU/17%PHEMA	10 or 15% of sol-densil
8	PU	–
9	IPN 83%PU/17%PHEMA	–
10	PHEMA	–
11	PHEMA	5% of nanosilica
12	PHEMA/water	–
13	PHEMA/water	10, 20, 30 or 40% of nanosilica

Nanocomposite NoNo 1–5 and 10–13 were synthesized by free radical thermal polymerization in the temperature range 70–110 °C in 2.0–2.5 ml syringes. The mixture for polymerization contained HEMA liquid monomer (Sigma-Aldrich), azobisizobutyronitrile as the initiator and triethylene glycol dimethacrylate as the crosslinking agent. For samples NoNo 3–5 and 12–13, the distilled water as a porogen was added into the polymerization mixture in the amount of 2/3 from the sample weight. BAC of different classes (Table 2) were introduced into the polymer matrix by three ways: directly into the matrix (samples NoNo 1 and 3), with the colloidal solution of nanosilica (sample No 5) and in form of sol-densil (samples NoNo 2 and 4). Samples NoNo 8–13 had no BAC in their content. Fumed nanosilica A-300 (pilot plant of the Chuiko Institute of Surface Chemistry, Kalush, Ukraine) with specific surface area 315 m^2^/g was heated at 400–450 °C for 4 h prior its use. The colloidal solution of nanosilica was prepared with the help of ultrasound. Sol-densil, which is a nanosilica with mechano-chemically immobilized on its surface BAC [14, 16], was dispersed before polymerization in the initial mixture by the help of ultrasound. Composite No 13 with a high content of nanosilica was specially synthesized for IR spectroscopic studies. All synthesized samples had cylindrical shape, different elasticity and transparency.

**Table 2:** BAC which were included into nanocomposites

Compound, manufacturer	wt%
Metronidazole (China)	3.6
Decamethoxin (“Pharmhim”, Ukraine)	2.9
ZnSO_4_ × 7H_2_O (Russia)	2.2
AgNO_3_ (“Macrokhim”, Ukraine)	1.3
Glycine (China)	2.2
Tryptophan (China)	2.9

Nanocomposites NoNo 6–9 based on PU or IPN 83%PU/17%PHEMA were synthesized by joint efforts of staff of the Institute of Macromolecular Chemistry and Chuiko Institute of Surface Chemistry of NAS of Ukraine [12, 14]. Samples had the form of films with thickness 1 mm.

The porous structure of the material after swelling in aqueous media was investigated by a confocal laser microscope LSM 510 META (Carl Zeiss, Germany), with ×600 magnification. Morphology of samples was studied by scanning electron microscope Nova NanoSEM 450 (FEI, Germany) with Schottky field emission electron source and low-vacuum SED (LVD) detector. Prior their investigation, hydrogels (samples NoNo 10–13) were freeze-dried in a vacuum for 24 h using installation Freeze dryer CoolSafe 110-4 PRO (Denmark), at a temperature of −110 °C.

The pore size in the samples were calculated by BET analyzer BELSORP-mini II (Japan).

IR spectra of initial PHEMA and products of its interaction with nanosilica (composite No 13) were recorded on a spectrophotometer Specord M80 (Carl Zeiss, Jena, Germany). For this purpose, samples were dispersed in an agate mortar, mixed with KBr (Aldrich) in a ratio of 1:50 and pressed into plates by size 5 × 20 mm.

An interaction of the HEMA with the nanosilica surface in aqueous medium was examined with the help of adsorption method. An equilibrium concentration of HEMA in solution was determined by back spectrophotometry after oxidation by potassium dichromate, *λ* = 440 nm [17]. Various spectrophotometric methods to determine the BAC concentration in solution were used to study their adsorption onto nanosilica (see below).

To study the release kinetics of BAC, samples of nanocomposites were dispersed in distilled water and the concentration of the released substance was measured in the medium through the regular time intervals. The concentration of ions Zn^2+^ and Ag^+^ was determined by extraction-photometric method based on the dithizone reaction in acidic medium [18, 19]. Metronidazole content was measured in the absorption maximum at the wavelength of 318 nm. Decamethoxin concentration was determined by the photocolorimetric method based on the eosin reaction [20]. Amino acids were defined by ninhydrin method [17]. The swelling rate of nanocomposites was calculated as attitude of increase of mass of sample (in the process of swelling) to initial mass and was expressed in percent.

Thermal properties and stability of samples were studied using derivatograph Q-1500D (Paulik, Paulik & Erdey, MOM, Hungary) with the computer data registration. TG were recorded in the temperature range from 22 to 974 °C. Samples with mass 50–80 mg were heated at 10 °C/min in air. Differential curves of DTA and DTG were recorded simultaneously.

## Results and Discussion

**Fig. 1:**
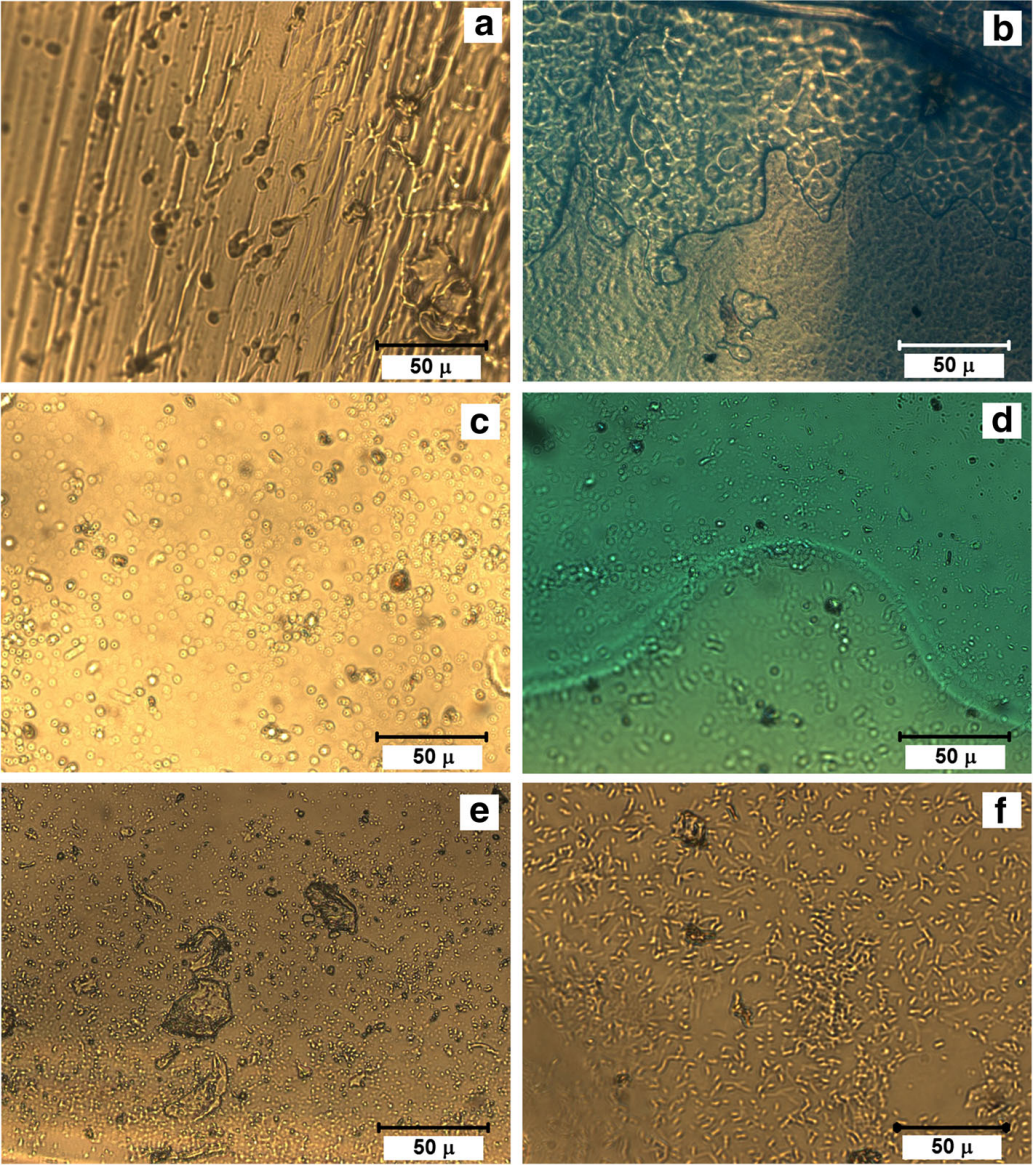
LSM samples: PHEMA (No 10)—dry (**a**) and swollen (**b**); IPN PU/PHEMA (No 9)—dry (**c**) and swollen (**d**); and PU (No 8)—dry (**e**) and swollen (**f**). Methylene blue solution is used in the samples (**b**) and (**d**) for the image contrast

The images of initial polymer matrices without filling in a dry form and after swelling in an aqueous medium obtained with LSM are shown in Fig. 1a–f. As expected, the pores were better visible in the swollen PHEMA. They had shape of channels and branchings in the micron range. The matrix structure based on PU and IPN 83%PU/17%PHEMA after exposure to water remained almost unchanged.

**Fig. 2:**
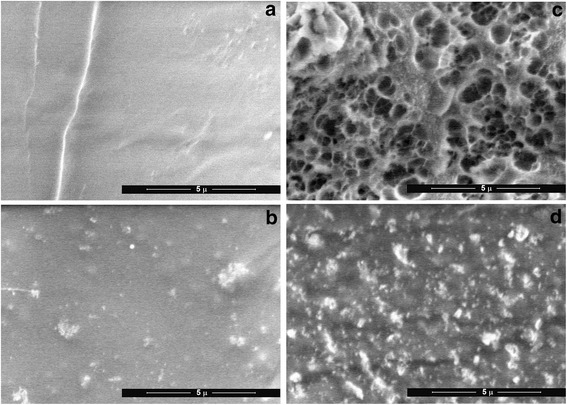
LVSEM of samples after freeze-drying: PHEMA (No 10) (**a**); PHEMA/5% nanosilica (No 11) (**b**); PHEMA synthesized in the presence of water (No 12) (**c**) and PHEMA synthesized with water/10% nanosilica (No 13) (**d**)

To study the structure of non-transparent materials based on PHEMA synthesized with water as a porogen, the SEM microscopy was used. The samples prior to imaging were freeze-dried. It was assumed that pores formed by water remained after freeze-drying. Indeed, LVSEM images show that the sample synthesized without porogen had a dense structure (Fig. 2b) while samples synthesized with water are porous with micron range size of pores (Fig. 2c). Samples filled with nanosilica demonstrated roughness of surface (Fig. 2b, d).

**Fig. 3:**
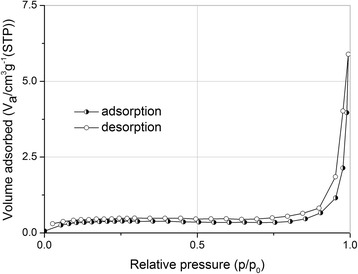
Nitrogen adsorption/desorption isotherm for freeze-drying PHEMA sample synthesized with porogen (No 12)

The porous structure of the sample No 12 was confirmed by low-temperature nitrogen adsorption technique. As it is shown in Fig. 3, isotherm has hysteresis at low pressure that testifies the presence of micro- and mesopores.

**Fig. 4:**
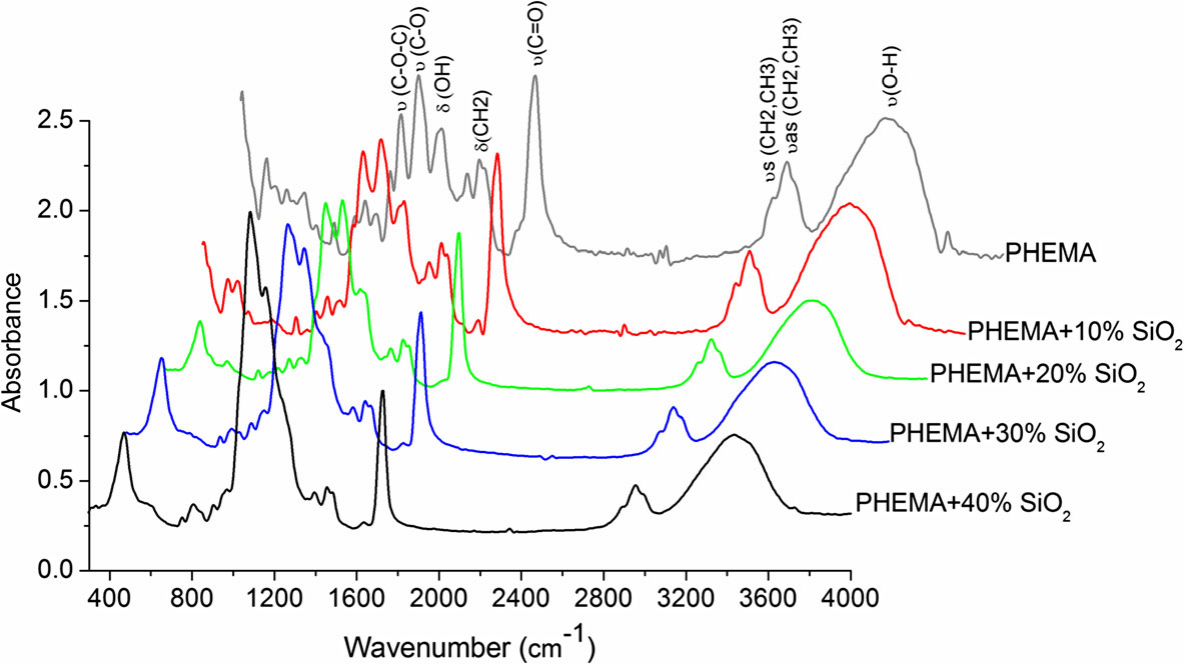
IR spectra of PHEMA/nanosilica composites (series No 13)

One of the possible reasons of slow BAC release from composite materials may be interaction of nanosilica filler with polymer matrix. Previously, by applying IR spectroscopy, we proved a possibility of hydrogen bond formation between the carbonyl group of HEMA monomer and silanol group of nanosilica surface [21]. In this work, we extended the study to the interaction of PHEMA with nanosilica filler. For this purpose, the samples of PHEMA with a high content nanosilica (series No 13) were synthesized and their IR spectra were investigated (Fig. 4).

In the obtained spectra, four regions which characterize the chemical structure of PHEMA are clearly visible: O–H stretching (3700–3000 cm^−1^); C–H stretching, corresponding to methyl and methylene groups (3000–2800 cm^−1^); C=O stretching (1770–1660 cm^−1^) and a fingerprint region (below 1500 cm^−1^). On the spectra, there are no bands of HEMA monomer including C=C double bond (1634 cm^−1^) and narrow band (3748 cm^−1^) which corresponds to free silanol groups of nanosilica. Whereas the intensity of the band which is responsible for carbonyl group of polymer does not change, and after decomposition by Origin 7.0 (OriginLab Corporation, Northampton, MA), it is symmetrical. These spectral data confirm the interaction of nanosilica surface with the polymer.

**Fig. 5:**
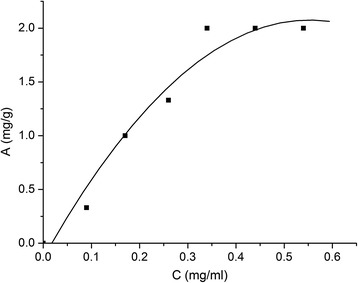
Adsorption isotherm of HEMA on the nanosilica surface from aqueous medium

As could be seen from the results of adsorption studies presented in Fig. 5 and Table 3, the adsorption isotherm of HEMA reaches plateau at the concentration 2 mg/ml. This concentration corresponds to adsorption density of three monomer molecules per 100 nm^2^ of nanosilica surface. In the presence of HEMA, the adsorption of BAC on nanosilica is reduced. Amino acids are not adsorbed at all.

**Table 3:** Effect of HEMA on BAC adsorption (*A*, mmol/g) on nanosilica surface

The initial content of BAC in the solution, milligrams per milliliter	Decamethoxin		AgNO_3_		Metronidazole		ZnSO_4_	
	*A*	*A*_HEMA_^a^	*A*	*A*_HEMA_	*A*	*A*_HEMA_	*A*	*A*_HEMA_
0.2–12.5	3	2.8	2.6	1.7	0.8	0.5	0.4	0.1
0.4–25	6.2	6.1	3.1	1.7	1.1	1	0.8	0.7

^a^ *A*_HEMA_—adsorption of BAC in the presence of solution of HEMA

**Fig. 6:**
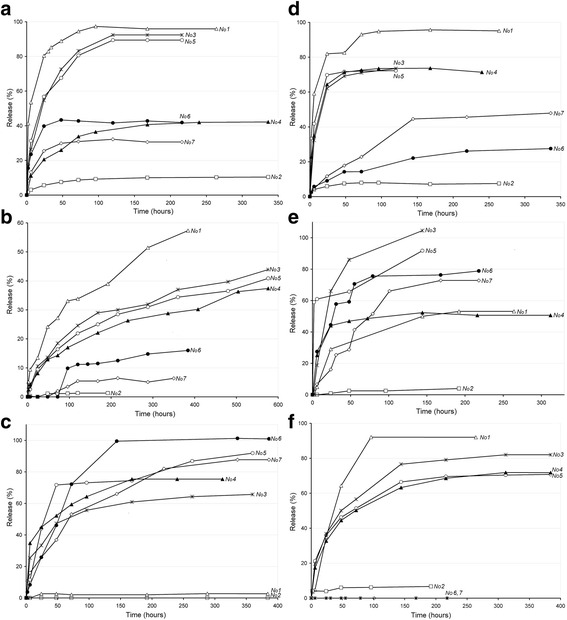
BAC release kinetics from nanocomposites: **a**) metronidazole, **b**) decamethoxin, **c**) Zn^2+^, **d**) Ag^+^, **e**) glycine and **f**) tryptophan. Curve numbers correspond to the samples in Table 1

Earlier, we studied the release kinetics of BAC from PU, IPN PU/PHEMA and PHEMA matrices [27]. In this work, the study of the BAC release from PHEMA-based materials synthesized with the porogen was continued. Analysis of the obtained kinetic curves showed a general pattern; in the case of materials synthesized without porogen, substances which were immobilized on nanosilica (as sol-densil) had significantly decreased release levels (Fig. 6, curves 2, 6, 7); a dramatic exception was observed in the case of immobilized zinc sulphate (Fig. 6c, curves 6 and 7). In addition, nanosilica materials swell up in lower extent. Apparently, the interaction of the filler with the polymer network leads to its compaction and the reduction of permeability. That was not the case for the materials synthesized with porogen for which the profiles of release from nanosilica-containing materials and without the filler were not too different (Fig. 6, curves 3, 4, 5). Thus, the pores in polymer matrix which increased during swelling practically eliminated inhibitory effect of nanosilica on the release of BAC.

Hence, we conclude that the combination of pore formation with filling of PHEMA matrix by nanosilica permits to synthesize nanocomposites with a certain degree (Table 4) and duration (Fig. 6) of BAC release. Introduction of BAC directly to the polymer matrix leads to a very rapid release (Fig. 6, curve 1), which is undesirable since our aim is to create materials with a prolonged effect. An additional factor of slowing the BAC release is the formation of the IPN PU/PHEMA (Fig. 6, curve 7). According to [22], crosslinked polymers demonstrate a lower release rate due to increasing barriers to diffusion and prolonged relaxation of polymer chains. Decamethoxin release is a particulary long-term process. Its kinetic curves even after 600 h are far from reaching the plateau (Fig. 6b). This behaviour can be explained by the pecularity of the decamethoxin structure. Its molecule contains two quaternary nitrogens as well as two space menthol fragments with a great distance between them, which contributes to its braking by polymer network and allows to interact with the maximal number of silanol groups of filler [23]. Surprisingly, we observed no tryptophan release from PU-containing matrices and extremely low (3%) release of Zn^2+^ ions from unmodified PHEMA (Table 4). In the first case, it can be explained by specific chemical interaction of tryptophan with PU during synthesis of composite. In the second case, the low release is probably due to the fact that hydrophilic PHEMA matrix keeps highly hydrated Zn^2+^ ions (hydration energy—2050 kJ/mol) forming hydrogen bonds while hydrophobic polyurethane chains, on the contrary, contribute to their repultion. The reason of low BAC release from composite PHEMA/sol-densil (sample No 2) is explained in Table 4.

**Table 4:** BAC maximal release (in %) from the samples

BAC or ion	Series numbers according to Table 1						
	No 1	No 2^a^	No 3	No 4	No 5	No 6	No 7
Metronidazole	96	10	92	42	89	42	31
Decamethoxin	57	1	44	40	41	16	6
Zn^2+^	3	0	66	75	92	98	86
Ag^+^	95	8	74	71	72	31	52
Glycine	42	4	100	52	92	79	73
Tryptophan	92	7	77	63	67	0	0

^a^According TGA, a small amount of sol-densil went into samples of this series (poor preparation technology)

During the first 1–2 days, an increase in the mass (swelling phase) then a gradual decrease (desorption phase) is observed. Comparison of the materials with identical content, but based on the different matrices, demonstrates that the samples with the IPN PU/PHEMA are more hydrophilic than the samples based on PU. Furthermore, for the materials based on PU, the weight of the samples over time becomes even lower (8–10%) than the weight of the initial sample. Swelling of the materials based on PHEMA is quite pronounced, especially in the samples synthesized with porogen. For example, PHEMA-based samples with zinc sulphate synthesized with porogen and without filler demonstrate the increase in their weight 2.6 times.

**Fig. 7:**
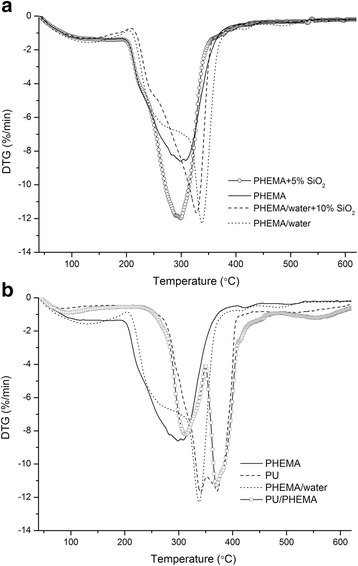
DTG curves of samples based on various polymer matrices with a filler (**a**) and without it (**b**)

The results of TG analysis are presented in Fig. 7. The DTG curve of PHEMA homopolymer shows a single step of reaction which is reflected as a single peak in the range 210–360 °C with a maximum rate of decomposition at 300 °C for PHEMA polymerized in bulk. We observed that addition of water as a porogen into the PHEMA matrix changes thermograms more significantly then filling by nanosized nanosilica. For example, at the DTG curve of PHEMA synthesized with porogen (Fig. 7a), the shoulder in range 240–310 °C appears and the peak shifts toward to 340 °C. It is believed [24] that the presence of water prevents the formation of intramolecular hydrogen bonds. This leads to stabilization of radical fragments and preventing of adverse reactions, such as cyclization of side groups. As the polymerization of HEMA proceeds, the growing polymer phase separates into the aqueous phase as droplets, which are joined together. Eventually, they are fixed in the form of a network of polymer chains surrounded by interconnected channels which are occupied by water. As a result, during polymerization in a solvent, more longer cross-linked macromolecular chains are formed, thus the thermal stability of matrix increases. It was found that IPN PU/PHEMA and PU matrices demonstrate most thermostability with the main stage of weight loss in the temperature range 290–400 °C. The DTG curve of IPN PU/PHEMA hybrid material (without any filling) contains two distinct mass loss peaks centred approximately at 310 and 370 °C, which correspond to the thermooxidative degradation of PHEMA and PU, respectively (Fig. 7b). These DTG data confirm the fact that in IPN PU/PHEMA material, each of the components forms a separated phase. After filling of IPN PU/PHEMA by nanosilica with immobilized BAC, the biphase structure of obtained nanocomposites remains.

Thus, the synthesized materials are enough thermostable and can be used at physiological temperature of 37 °C and suitable for heat sterilization.

## Conclusions

Different composite materials were obtained modifying PHEMA by introducing nanosilica (densified and nanosized), creating IPN PU/PHEMA and forming pores. These materials were characterized by controlled release of deposited BAC and may be useful for manufacturing of medical devices.

For the materials synthesized without porogen, significantly diminished release of BAC which were immobilized on nanosilica (as sol-densil) were observed. We explain such diminished release of BAC by the interaction of nanosilica with PHEMA which makes the matrix denser and less permeable. In contrary, for the composites which were synthesized with porogen, the release profiles for nanosilica containing materials and with no nanosilica were quite similar. Thus, the pore formation in polymer matrix practically eliminated the inhibitory effect of nanosilica on the release of BAC.

By applying DTG method, it was found that addition of water as porogen increases thermal stability of the polymer matrix more than the introduction of nanosilica. A biphase structure of IPN PU/PHEMA matrix was proved. The DTG results were generally correlated with the release kinetics of BAC.

## Abbreviations

BAC: Biologically active compounds
DTA: Differential thermal analysis
DTG: Differential thermogravimetry
HEMA: 2-Hydroxyethyl methacrylate
IPN: Semi-interpenetrating networks
IR: Infrared
LSM: Laser scanning microscopy
LVSEM: Low-vacuum scanning electron microscopy
PHEMA: Poly(2-hydroxyethyl methacrylate)
PU: Polyurethane
